# Refinement of Outcome Bias Measurement in the Parental Decision-Making Context

**DOI:** 10.5964/ejop.v15i1.1698

**Published:** 2019-02-28

**Authors:** Kaja Damnjanović, Sandra Ilić, Irena Pavlović, Vera Novković

**Affiliations:** a Laboratory for Experimental Psychology, Department of Psychology, Faculty of Philosophy, University of Belgrade, Belgrade, Serbia; University of Belgrade, Belgrade, Serbia

**Keywords:** outcome bias, parents, involvement, decision domain, health decisions, neutral position evaluation

## Abstract

The aim of this study was twofold: one was to test the impact of the involvement on the parental outcome bias, and the second was to refine the measurement of outcome bias, normally reported as the difference between evaluations of a single decision, with different outcomes assigned to it. We introduced the evaluation of a decision without an outcome, to induce theoretically normative evaluation, unbiased by outcome, from which the evaluation shift could be calculated in either direction. To test this refinement in the parental decision-making context, we produced childcare dilemmas with varying levels of complexity, since the rise of complexity induces stronger bias. Complexity was determined by the particular combination of two factors: parental involvement in a decision - the amount of motivation, interest and drive evoked by it – and whether the decision was health-related or not. We presented parents with the decisions for evaluation, followed by a positive and a negative outcome, and without an outcome. The results confirm the interaction between involvement and domain on decision evaluation. Highly involving decisions yielded weaker outcome bias than low-involvement decisions in both health and non-health domain. Results also confirm the validity of the proposed way of measuring OB, revealing that in some situations positive outcomes skew evaluations more than negative outcomes. Also, a highly-involving dilemma followed by negative outcome did not produce significantly different evaluation compared to evaluation of a decision without outcome. Thus, adding a neutral position rendered OB measurement more precise and our involvement-related insights more nuanced.

## Involvement in a Decision

Decision making is a fundamental cognitive process which occurs on a daily basis. People have to make decisions about what to wear, whether to accept a new job position and move to a different city, or whether to vaccinate their child or not. A very basic feature by which all of these decisions differ is the domain of life in which people are forced to decide. When making decisions, not only are people differentially sensitive to experimental manipulations of stimuli from different domains, such as health, money, morals, shopping etc., but they also use different *strategies* when making decisions in different domains (Goldstein & Weber, 1995). The domain in which a decision maker (DM) is expected to decide, also determines the importance of the decision (Damnjanović, 2013; Gvozdenović & Damnjanović, 2016; Tanner & Medin, 2004; Tanner, Medin, & Iliev, 2008). The importance of a decision shapes the level of *involvement* in that decision. Involvement is defined as a state of motivation, arousal or interest regarding an activity or an object (Damnjanović, Graeber, et al., 2018; Rothschild, 1984), or as an internal state variable that indicates the amount of motivation, interest and drive evoked by a stimulus or situation (Bloch, 1982; Jevtović & Damnjanović, 2015; Mitchell, 1981, 1979). In behavioral terms, the level of involvement pertains to the level of the energy spent, the number of alternatives examined, and the extent of the decision-making process (Engel & Blackwell, 1982). In line with this, decisions vary on a continuum from quite ordinary and unexceptional to those that require considerable thought and induce higher levels of involvement, reserved for issues or objects that carry great meaning for the DM (Solomon, Bamossy, Askegaard, & Hogg, 2006). One of the decision-making domains that stands out for its high level of importance is the domain of health (Wroe, Turner, & Salkovskis, 2004; Zikmund-Fisher, Sarr, Fagerlin, & Ubel, 2006). In the health domain itself, certain decisions are more important and involving than others, depending on their specific content (Thompson, 2007). Deciding whether or not one should give Vitamin C to their child to prevent a cold is important, but less so than deciding whether one should engage in similarly preventive behavior such as vaccinating one’s own child.

## Parental Involvement in a Decision

The level of involvement in the decision differs depending on DM’s role relative to the object of the decision (Arora & McHorney, 2000; Donovan & Jalleh, 2000); that is, does the DM serve a proxy, or not. Deciding whether one's *own* child should be vaccinated is more involving than deciding whether *some* or *all* children should be vaccinated. The level of involvement is therefore influenced by decision content (e.g. vitamin C or a vaccine), the role of the DM relative to the object of the decision (e.g. a parent of the object), and the object itself (e.g. a DM’s child) (Gvozdenović & Damnjanović, 2016; Liu, Polman, Liu, & Jiao, 2018; Polman & Emich, 2011; Wang, 1996a, 1996b). Findings from the field of rationality in decision making which focus on the decision maker provide empirical support for this stance: DMs decision (e.g. risky choices) depends on what he/she is deciding about (e.g. money or health; Wang, 1996a, 1996b); is DM deciding for him/herself, or is DM a proxy (Liu, Polman, Liu, & Jiao, 2018); how big is the psychological distance from the object, e.g. did DM earn money he is gambling with or did he get it by chance (Damnjanović, 2013; Polman & Emich, 2011). On top of all of that, and in the accordance with the concept of the intensive parenthood (Arendell, 2000; Smyth & Craig, 2017) parents, for one reason or another, share some preconceptions about not only specifically their own children, rather about children in general.

Decision making performed by parents for their children has some distinct aspects, like specific involvement, or the inherent proxy role of the DM. For example, in the contemporary societal context of intensive parenting (Arendell, 2000; Smyth & Craig, 2017), parents are encouraged to take an active role in their children’s healthcare (Pyke-Grimm, Degner, Small, & Mueller, 1999), which places an even greater responsibility on them, due in part to the number of choices available (Wagenaar, Keren, & Lichtenstein, 1988). Parents have to make decisions regarding matters in which they often have little to no expertise while receiving various information from sources of varying levels of credibility (Chaudry, Henly, & Meyers, 2010, Hussain, Ali, Ahmed, & Hussain, 2018). In other words, parents who have to make a decision about their child’s care might face cognitive obstacles during the decision-making process, which might hinder them in making a truly informed choice. Hence, shedding light on the level of influence decision domain and involvement exert on parents when they are deciding about their children, and the way their decision-making strategies are affected by the context of said decision could offer valuable insight for understanding and potentially providing solutions for the various issues which abound in childcare decisions.

## Outcome Bias

In addition to the extent of information processing required for highly involving choices, the decision-making process is further complicated by patterns of deviations from normatively defined rationality, namely cognitive biases (Solomon, Bamossy, Askegaard, & Hogg, 2006). As choice complexity rises, people tend to simplify decision-making processes by relying on simple heuristics (Payne, Bettman, & Johnson, 1988, 1993). According to normative models of rational decision making, differentiating between a good decision and a positive outcome should be the basis of every decision-making analysis (Baron & Hershey, 1988). However, people tend to judge decisions with negative outcomes as being of significantly lower quality compared to the same decisions resulting in positive outcomes. For example, because one of the main components contributing to vaccine hesitancy is the circumstantial, temporal connection between vaccination and various health issues which occur in early childhood (Salmon, Dudley, Glanz, & Omer, 2015), one of the possible reasons why parents might be hesitant to vaccinate their children (either at all or according to an official schedule) is that the decision about vaccination is strongly focused towards its outcome (Goldenberg, 2016; Miton & Mercier, 2015), and considered good or bad based on the consequences which are *ostensibly* but not *actually* connected with it. The systematic tendency to evaluate decisions based on their outcomes is called outcome bias (OB) (Baron & Hershey, 1988). From the perspective of normative theories of reasoning, outcome-based evaluations are considered irrational, due to the normative request that the order of preferences must be immune to the descriptions of the outcomes or the situations (Von Neumann & Morgenstern, 1945). Contrary to this, different reasoning patterns stemming from zones of gains and losses are postulated in the value function of the prospect theory (Tversky & Kahneman, 1982, 1992), or in concepts of appetitive and defensive motivation, linked with goal-directed and goal-determined behaviors (Lang & Bradley, 2013). Finally, from the perspective of the theory of ecological rationality, which posits that some deviations from normatively defined rationality are actually rational in particular environments, the aforementioned biased evaluations might be adaptive in specific contexts (Gigerenzer, 2008; Gigerenzer & Brighton, 2009). The main question this theory poses is which context will serve to facilitate *adaptive* use of judgments deviating from the normative.

## Measurement of the Outcome Bias

Aside from the opposing theoretical perspectives that consider biased reasoning to be an adaptive feature versus ion or a bug, another issue is the measurement of cognitive biases, that is – *what* is a biased estimation or decision deviating *from*. Namely, OB is usually measured in an evaluation task standardly used in the judging and decision-making paradigm. Participants are presented with the same decision once followed by a positive and once by a negative outcome and asked to evaluate the decision, *regardless of the outcome*, most often on a scale (e.g. ranging from 1 - very bad to 7 - very good) (Baron & Hershey, 1988). When the calculated difference between mean evaluations of decisions followed by the positive and the negative outcomes is statistically significant, researchers deem that OB has occurred (Baron & Hershey, 1988; Damnjanović, Graeber, et al., 2018, Mazzocco, Alicke, & Davis, 2004; Sezer, Zhang, Gino, & Bazerman, 2016). As is the case with some other cognitive biases (e.g. framing effect), such a measuring technique is not quite correspondent with the theoretical model it stems from. Namely, if the outcome, or both the outcome and consequences of a given decision *are always presented* and indeed turn out to skew subjects’ evaluations, such task design renders the measurement of outcome bias quite imprecise and our conclusions drawn from it rather limited. For example, if the participants, after being presented with the decision followed by a negative and then by a positive outcome, evaluate these decisions on a scale from 1 (the worst possible decision) to 7 (the best possible decision), by marking 1 and 3, respectively, we say that they were biased by the outcomes of those decisions. However, what can we conclude from this difference, aside from the difference itself? What did their evaluation deviate from? If, for example, we assume that marking the number 4, that is - the middle position (a decision is neither good nor bad) is the normatively correct answer, marking 1 after the negative outcome and 3 after the positive outcome both differ from this position, but in the same direction. In other words, in this case we cannot claim that our participants were biased by the outcome, seeing as how biased evaluation comprises deviation from the normative position. However, as some decisions are considered good or bad to start with, the normatively correct evaluation, not biased by outcome, does not have to be the middle position. This unbiased evaluation is missing from most outcome bias studies, because the participants never get the chance to evaluate (as good/bad or neither good nor bad) a decision prior to or without knowing its outcome. In other words, a missing component in OB studies is a true "control situation", i.e. a situation in which we might learn the "base" value of a certain decision. This base value can be determined by asking participants to evaluate a decision without a presented outcome (or consequence). Applying this procedure to OB measurement might answer a few largely neglected questions: what is the normatively correct answer, to what extent does the outcome skew evaluations and which ones, and are the participants’ evaluations skewed more by negative or positive outcomes? This new approach to measuring OB can provide insight into the base value of a certain decision and relative contribution of positively and negatively valenced outcomes to the DM’s evaluation shift which would add on to the existing descriptive theories of reasoning.

The present study had more than one aim. First, we aimed to demonstrate that the strength of the OB is determined by the level of the DM’s involvement in the decision, which stems from his/her role relative to the object of the decision, the domain, and the content of the decision. Accordingly, we expect that high-involvement situations in which participants (who are also parents) evaluate a decision pertaining to highly important health content, made by a fellow parent and regarding a child, will yield stronger OB, in comparison to their evaluations in low-involvement situations.

In order to overcome the challenge set before commonly used measurements of outcome bias, we introduced the new approach to measuring outcome bias. By establishing the reference point for further comparison between evaluations of decisions with positive and negative outcomes, the measurement of OB susceptibility could be refined. We expect that the relative effect of a positive outcome will be weaker than that of a negative outcome.

Altogether, these findings should aid in examining the parental decision-making process regarding their child in different contexts and understanding if and how this process is skewed across various domains.

## Method

### Sample

For calculating the sample size the power analysis was used. The probability of detecting a statistically significant outcome bias effect by a bivariate *F*-Test (at level *p* < .01) of the effect size of .7, as reported by Baron and Hershey (1988), for a sample of 20 subjects per experimental group amounts to 99.9%.

The final sample comprised 46 parents of children under the age of 7, as the order of the presentation of tasks was counterbalanced two groups were formed. Participants were recruited via the Internet by using the combination of posting the links to experiments on parental Facebook groups and the snowball method. The final sample comprised only subjects who participated in both experimental sessions. Dropout rate between sessions was 29%, that is - 65 parents took participation in the first session, while 46 parents participated in both sessions. The mean age was 37 (*SD* = 3.368); 76% of the sample was female.

As an incentive for the participation all participants were told that, provided they participate in both experimental sessions, they will be included in a lottery in which two participants will be randomly selected and awarded a book published by the University of Belgrade.

### Materials

For the purposes of measuring the susceptibility to outcome bias, evaluation tasks were used. Each task was a vignette and consisted of a prologue - a description of a situation that contains a dilemma, and the option the decision maker opted for, which was or wasn’t followed by the either positive or negative outcome of that decision. Participants in the main experiment were instructed to evaluate the presented decision on a scale from 1 (the worst possible decision) to 7 (the best possible decision).

Prior to the construction of the tasks, a focus-group discussion with 9 parents, members of the PTA in a nearby preschool facility, was organized. First, they listed situations in which a child-related decision occurs and that they or other parents encounter. Together, they listed 24 situations. Then, they individually categorized those situations as high involving or low involving. The definition of involvement has not been provided for parents, rather they conveyed the subjective meaning of it, usually by the time spent for deliberation and by potential consequences of the decisions. With the aim of constructing reliable pairs of tasks, a pilot study was conducted. From every situation, two evaluation tasks were constructed (with positive and with negative outcomes). Other group of parents (*N* = 50), recruited via teachers in preschool facility, of children younger than 7 (mean age: 36.19, *SD* = 6.69, 74% female) evaluated decisions in 24 pairs of tasks with various content, in a within-subjects design, in two experimental sessions. The design was 2 (role of the DM: parents and non-parents as protagonists) x 2 (outcomes: positive and negative). The dependent variable was evaluation of each decision, made by participants on a 7-point scale (1 – the worst possible decision, 7 – the best possible decision). The measure of the outcome bias was average differential score calculated as the difference between estimations of situations with opposite outcomes. The registered difference between evaluations of decisions followed by opposite outcomes was significant for each pair of tasks - that is each pair of tasks yielded outcome bias. The sizes of the main effects of the outcome factor (Cohen's *d* coefficients) for each pair of stimuli were ranging from .223 to 1.604, with mean value of .957. The effect sizes served as the criterion for selection of the tasks for the main experiment.

In the final twelve tasks used in the main study (Appendix), each dilemma described was either non-health or health related, with high- or low-involvement content. All structural aspects of the tasks had been made uniform: they all contained a nameless set of parents, who were making decision about a nameless child’s care under advisement from an authority figure, and who made the decision to abstain from the advised action which was followed by a positive or a negative outcome, or no outcome at all.

### Design

The study employs a 2 (domains of the decision: health and non-health) x 2 (levels of involvement: high and low) x 3 (outcomes: positive, negative and without outcome) repeated design. The involvement and domain factors are both within-subjects factors. The intersection of the levels of these two factors results with the following 4 dilemmas: high-involvement health decision (vaccination), low-involvement health decision (car sickness), high-involvement non-health decision (first grade application), and low-involvement non-health decision (recreational sports). The outcome factor is also a within-subjects factor, with the following levels: positive outcome (PO), negative outcome (NO) and without outcome (WO). In total, there were twelve tasks: 4 dilemmas x 3 outcomes.

The dependent variables (DV) were susceptibilities to outcome bias measured in two different ways. The first DV was the traditionally used measure of OB, calculated as the mean difference between evaluations of PO and NO decisions. The second and the third DV were calculated by using the aforementioned newly proposed way of measuring susceptibility to OB and represented the differences between average evaluations of PO and WO decisions (positive shift) and NO and WO decisions (negative shift).

### Procedure

The experiment was conducted via an online questionnaire created using Google Forms platform. In the first week of experimentation, half of the participants received a questionnaire containing a set of 4 WO tasks, as well as two sets of 2 PO and 2 NO tasks, while the other half received a questionnaire containing the same 4 WO tasks, and two sets of 2 PO and 2 NO tasks counterbalanced against the first half’s questionnaire. Participants were instructed to carefully read the text of each task presented to them and to evaluate the described *decision* on a 7-point scale (1 – the worst possible decision, 7 – the best possible decision). Participants were then asked to provide their e-mail addresses in order to receive the link to the second questionnaire. It has been emphasized that their e-mail addresses will not be in any way connected to the data they have provided us with, or used for the purposes other than enabling participation in the second session. After a week, they received the link to the second questionnaire, which contained only 4 tasks: 2 PO and 2 NO counterbalanced against the PO and NO tasks they completed one week earlier. The instructions remained unchanged. Personally generated codes were used for matching data from two sessions. All participants signed a consent form prior to participation and were debriefed once the experiment was completed.

## Results

### Factors of the Outcome Bias

In the first step of the analyses, for measuring whether there was a significant difference registered between evaluations of decisions with opposite outcomes the paired samples *t*-test was used. As the dependent variables, two new variables were formed, both calculated as average evaluations of all decisions followed by only positive and by only negative outcomes. The *t*-test revealed that the difference between average evaluations of all the decisions with positive and with negative outcomes was significant, that is, the outcome bias was registered on the level of the total sample, *t*(45) = 11.474, *p* = < .001, *d* = 1.692. For determining whether the effect of the outcome, that is outcome bias, was registered on each of the tasks a series of four pairwise *t*-tests was performed. Participants’ evaluations of decisions followed by positive outcome and by negative outcome for each dilemma were used as dependent variables. The average evaluations of the decisions described in tasks, standard deviations, confidence intervals, *t*-test and Cohen’s d statistics for outcome bias obtained from the 4 experimental situations (2 domains x 2 involvement) are presented in Table 1. The analysis showed that significant outcome bias was registered on all four dilemmas. However, the effect sizes of the outcome factor were different for each dilemma. The lowest standardized difference between mean evaluations of decisions with opposite outcomes was observed in the vaccine related situation (health related high involvement), while the outcome had the greatest effect on the evaluations of sports related decision (non-health related low involvement).

**Table 1:** Group Differences in Evaluations of the Decisions With Positive and Negative Outcome in Four Experimental Dilemmas of Decision Making

Task (dilemma)	Domain x Involvement	Outcome	*M*	*SD*	99% CI		*t*	Cohen's *d*	*p*
					LL	UL			
Sport	Non-health, low						8.231	1.214	< .001
		Positive	5.196	1.558	4.58	5.81			
		Negative	2.848	1.349	2.31	3.38
School	Non-health, high						6.626	.977	< .001
		Positive	5.000	1.687	4.33	5.67			
		Negative	3.217	1.474	2.63	3.80
Travel	Health, low						6.324	.932	< .001
		Positive	4.826	1.495	4.23	5.41			
		Negative	3.000	1.265	2.49	3.50			
Vaccine	Health, high						2.949	.435	.005
		Positive	1.761	1.058	1.34	2.18			
		Negative	1.261	.535	1.05	1.47

*Note. N* = 46; CI = confidence intervall; LL = lower limit; UL = upper limit.

**Figure 1:**
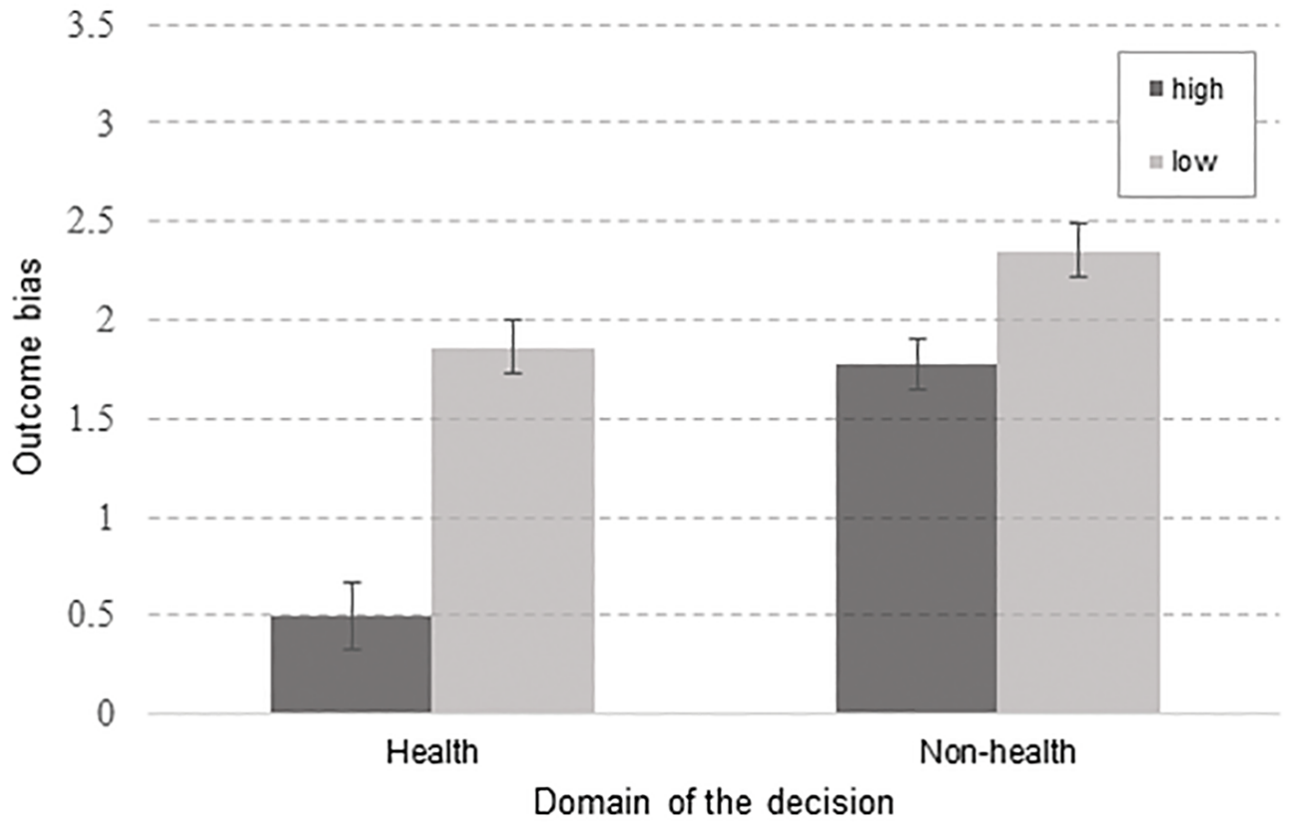
Interaction between involvement and domain. *Note.* y-axis maximum is 3.5, since the range of the OB (measured as differential score between estimations of decisions with opposite outcomes) does not exceed 2.5 points; high – high-involvement decision, low – low involvement decision.

A repeated measures ANOVA was conducted in order to test for differences in outcome bias yielded by different pairs of tasks, that is – the dilemma factor. As dependent variables four new variables, one for each dilemma, were created, each calculated as the difference between evaluations of decisions with opposite outcomes. The significant difference in outcome bias was registered between different levels of the dilemma factor, *F*(2.051, 92.294) = 9.923, *p* < .001, η^2^ = .181^i^. A two-way (health, non-health) x involvement (high, low) ANOVA for repeated measures was run to examine the effects of the involvement and the domain of the decision on the outcome bias. Again, as the dependent variables differences between evaluations of decisions with opposite outcomes for each dilemma were used. The analysis of variance revealed that the interaction between the effects of the involvement and the domain of the decision on evaluation of decisions was significant, *F*(1, 45) = 7.203, *p* = .01, η^2^ = .138. Significant main effects of the involvement, *F*(1, 45) = 15.159, *p* < .001, η^2^ = .252, as well as the domain, *F*(1, 45) = 7.562, *p =* .009, η^2^ = .144, on participants evaluations of decisions were also registered. Post hoc tests using the Bonferroni correction revealed that participants evaluations were significantly higher for low involvement decisions (*p* < .001), with the mean difference between low and high involvement decisions of .946. Analysis also showed that participants evaluations for non-health related situations were significantly higher compared to health related situations (*p* = .009), with mean difference of .902. Simple main effects analysis showed that in the domain of health, highly involving decisions were yielding significantly weaker outcome bias than low-involvement decisions, *F*(1, 45) = 19.351, *p* < .001, and the effect had the same direction in non-health situations, *F*(1, 45) = 4.747, *p* = .035, albeit with significantly less influence. Strongest influence of the outcome was observed in low-involving non-health dilemma, while the weakest was registered in high-involving health dilemma (Figure 1). In short, subjects were more prone to be biased toward the outcome when evaluating less involving dilemmas.

### Factors of the Evaluation

**Figure 2:**
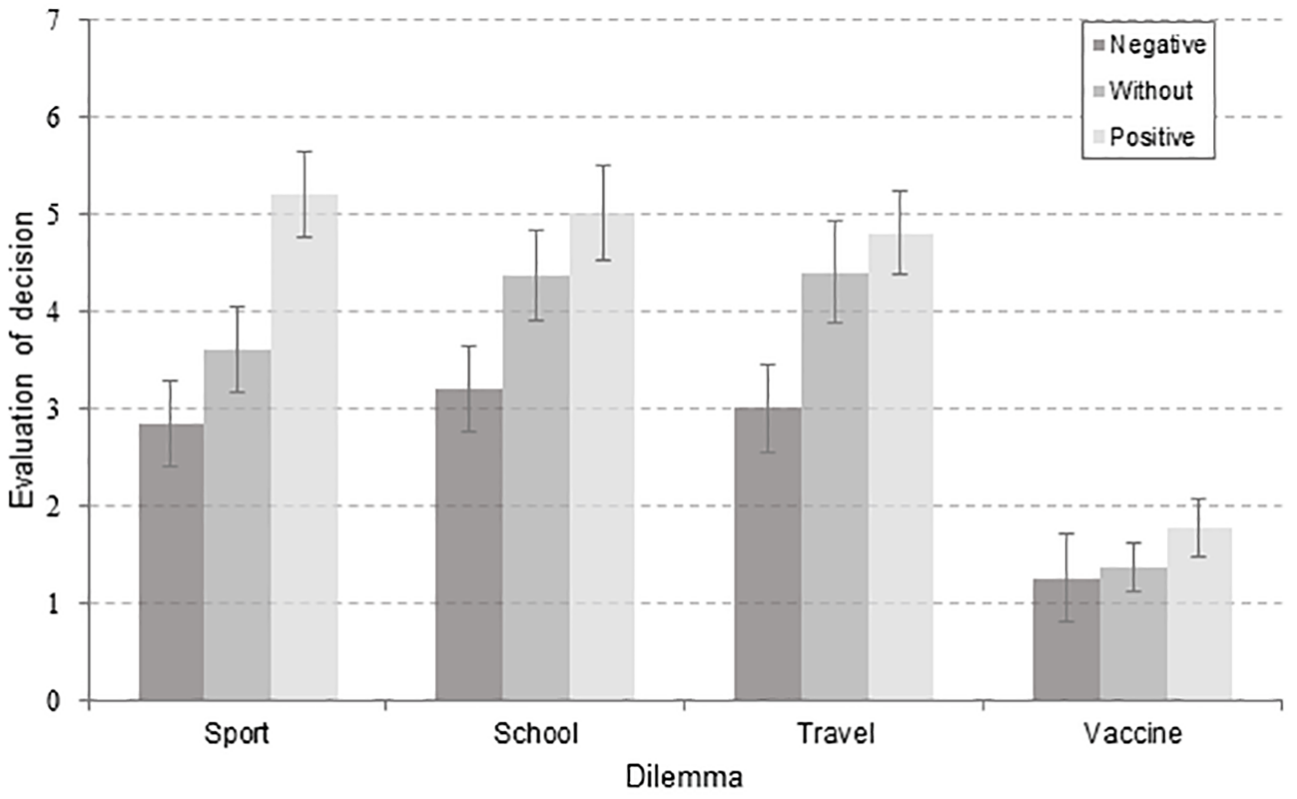
Evaluations of the decisions as the function of the outcome level in all four dilemmas. *Note.* Negative = negative outcome; Without = without outcome; Positive = positive outcome.

In the second step, with the aim of further decomposing the outcome bias effects and to describe the dynamics of the evaluations in more detail, evaluations of the decisions were again used as dependent variables, but the outcome factor had three levels: positive, negative, and without outcome. Second factor in the analysis was the dilemma, with four levels (sport, school, vaccine, travel) stemming from the intersection of levels of the involvement and the domain factors. A repeated-measures ANOVA was used to examine the effect of the direction of the outcome (3 levels: positive, negative, without outcome) and the dilemma (4 levels) on evaluations of the described decisions. The interaction between the two factors was significant, *F*(3.661, 164.765) = 8.074, *p* < .001, η^2^ = .152^ii^. Also, both the outcome, *F*(2, 90) = 74.621, *p* < .001, η^2^ = .624, and the dilemma, *F*(3, 135) = 95.343, *p* < .001, η^2^ = .679, had significant main effects on participants’ evaluations. Post hoc tests using the Bonferroni correction revealed that participants evaluations were significantly lower for decisions followed by negative outcomes compared to decisions without outcomes (*p* < .001), with the mean difference of -.853. The same goes for pairwise comparison between decisions without outcomes and decisions followed by positive outcomes (*p* < .001), with mean difference of -.761. However, pairwise comparisons of mean evaluations of different dilemmas, using the Bonferroni correction showed that the mean difference of -.123 between evaluations of travel and school dilemmas, .188 between evaluations of travel and sport dilemmas, and .312 between school and sport dilemmas were not statistically significant (*p* = 1.000, *p* = 1.000, *p* = .624, respectively). Pairwise comparisons of the interaction of these two factors, again with the Bonferroni correction, showed that participants’ evaluations of decisions were differently biased by opposite outcomes^iii^. In the vaccination dilemma only the positive outcome made statistically significant shift from the neutral position, that is – participants evaluated decision with positive outcome as better than the decision without outcome with the mean difference of .391 (*p* = .043). This was not the case with the decision followed by negative outcome, as the mean difference between evaluations of the decision with negative and the decision without outcome was not significant (*p* = 1.000) and amounted to .109. In both travel and school dilemmas only the negative outcome produced statistically significant shift from the evaluations of the same decisions presented without outcomes. For travel related dilemma the mean difference between evaluations of the decision with negative and without outcome was 1.391 (*p* < .001), while for school related dilemma it amounted to 1.152 (*p* < .001). When it comes to the positive shift from the neutral position, the mean difference between decisions with positive and without outcome for travel related dilemma was not significant (*p* = .071), and amounted to .435, and the same goes for the school related dilemma – mean difference was .630 (*p* = .075). Only in the sport related dilemma both the positive and the negative outcome induced significantly different evaluations of decisions, that is - the outcome bias was registered in both directions. Namely, participants evaluated sport related decision with negative outcome as significantly worse than the same decision without outcome, with the mean difference of .761 (*p* = .001), and the decision without outcome as worse than decision with positive outcome, with the mean difference of 1.587 (*p* < .001). The simple main effects analysis showed that the direction of the outcome had significant simple main effect on three out of four levels of situation factor, in sport, *F*(2) = 55.804, *p* < .001, school, *F*(2) = 31.777, *p* < .001, and travel, *F*(2) = 35.385, *p* < .001, dilemmas, but outcome did not affect the evaluations of the vaccine decision (*p* < .07). The dilemma had significant impact on all three levels of the outcome factor, for evaluations of decisions with positive, *F*(3, 150) = 103.350, *p* < .001, negative, *F*(3, 150) = 31.040, *p* < .001, and without outcome, *F*(3, 150) = 78.870, *p* < .001. Evaluations of decisions in four dilemmas for each level of outcome are shown in Figure 2.

Overall, direction of the outcome influenced the evaluations of decisions in the expected manner: all the decisions with negative outcome were estimated as worse (average *M* = 2.58, *SD* = 1.16) than those without stated outcome (average *M* = 3.44, *SD* = 1.46, 99% CI [2.86, 4.02], and those were also evaluated as worse than decisions with positive outcome (average *M* = 4.19, *SD* = 1.45, 99% CI [3.61, 4.77]).

In addition to the direction, the proposed novelty in assessing the outcome bias enabled analyzing the valence of the influence of the outcome. The valence of the influence was calculated as the differential score between situations with outcome, on one side, and the situation without outcome, on the other side. Therefore, there were two dependent variables in this step. First one was *the negative shift*, calculated as the difference between evaluations of decisions without outcome and with negative outcome. Second dependent variable was *the positive shift*, calculated accordingly. Again, interaction between the content and the shifting was significant, *F*(3, 135) = 4.677, *p* = .004, η^2^ = .094. Negative outcome produced bigger shifting from the neutral position than the positive outcome in school and travel dilemmas. In sports and vaccine dilemma the estimations were affected in opposing manner (see Figure 2).

## Discussion

Parents decide on behalf of their offspring on a daily basis, with varying levels of success, involvement, and rationality. But what would be the norm for rationality in these high-stakes decisions? Where do parents put their trust and what is an anchor for the decision? For example, the decision about vaccination can be viewed as a socially forced choice, due to the existence of two conflicting, but intense and explicit societal influences, in both the normative-medical and lay-experience form (Damnjanović, Graeber, et al., 2018). Cognitive system, in terms of imprint, does not necessarily discriminate between the two. So, what would be a rational decision from parental point of view? The normative model of rationality proposes that the sequence of our preferences should be invariant to the description of the preference (Von Neumann & Morgenstern, 1945). This means that mental representations of the preference, e.g. to vaccinate or not to vaccinate, should be ordered neatly in our cognitive system. One way of measuring this feature is to observe patterns of cognitive biases in parents.

### Factors of the Outcome Bias

In our research, we started with a few assumptions about domain, content and involvement in a decision which will briefly be outlined. A decision’s importance varies depending on the life domain about which a person is deciding (Gvozdenović & Damnjanović, 2016). Although the domain of health, and decisions belonging to its scope, showed to be of great importance (Wroe et al., 2004; Zikmund-Fisher et al., 2006), all health-related decisions are not of equal importance, which depends on the decision’s content. The content, along with the object of the decision (e.g. a child) and the decision maker’s role relative to said object of the decision (e.g. a parent to a child), affect the extent of the decision making process, i.e. the involvement in the decision (Arora & McHorney, 2000; Donovan & Jalleh, 2000; Thompson, 2007). The level of involvement in the decision further affects the level of the decision’s complexity, which induces systematic patterns of deviations from rationality (Damnjanović, Graeber, et al., 2018). Namely, when the complexity of the decision-making process rises, people tend to rely more on simple heuristics (Payne, Bettman, & Johnson, 1988, 1993). Because health-related decisions are also inherently strongly focused towards their outcomes or consequences (Goldenberg, 2016; Miton & Mercier, 2015), one of the ways to simplify decision-making processes is by neglecting the difference between a good decision and a positive outcome - that is, to make judgments which are biased by the valence of the decision’s outcome. However, our results do not confirm these assumptions.

Firstly, highly involving decisions yielded significantly weaker OB as opposed to decisions which were presumed to require lower levels of involvement. When presented with highly involving decisions from both health and non-health related domains, parents’ judgments were more resistant to the influence of the outcomes than when presented with health and non-health related decisions, which induce lower levels of involvement.

Secondly, and contrary to our expectations, parents from the sample were more prone to making judgments biased by outcome when judging about less involving as well as non-health-related dilemmas. Participants generally judged non-health decisions as better, compared to health-domain decisions, with the strongest OB observed in the low-involving non-health situation, and the weakest in the high-involving health situation, which is in line with the notion that some decisions of particular importance are not as susceptible to the impact of external influence. When people are deciding about something less involving, they are more interested in outcomes, rendering their judgment prone to bias. On the other hand, assigning highly involving decisions to the good or bad categories is not based on consequences, but on moral reasoning (Tanner & Medin, 2004; Tanner, Medin, & Iliev, 2008). This finding, that parents show less biased evaluations of highly involving decisions made by fellow parents also indicates that there might be some sort of “parental empathy” moderating our results.

### Factors of the Evaluation

The second and equally important aim of this research was gaining further insight into the dynamics outcome effects on decision evaluations through the aforementioned approach to measuring OB. The new approach includes evaluations of decisions in 'neutral situations', and comparison of evaluations across the three levels of situations. This measurement shift indeed revealed some details that were not apparent with the usual way of measuring OB, and allowed us to examine and map certain regularities in irrational judgements.

Negative vs. positive shift. When participants are presented with negative outcome, the difference between this evaluation, and the evaluation of a decision without outcome is greater than the difference between evaluations of decisions with a positive outcome and without any outcome in half of the tasks. In other words, negative outcomes have a stronger effect and tend to skew the participants’ judgments more, compared to the neutral situation in high involvement non-health related (school) and low involvement health related (travel) dilemmas. This particular shift is in accordance with our expectations, and with the prospect theory value function, which proposes the very same regularity about risky decision making: loss aversion (Kahneman & Tversky, 1979). In the context of our research, decisions followed by losses are deemed as poorer and as such, should be avoided. In line with the ecological rationality perspective, as well as the concepts of appetitive and defensive motivation, linked to goal-directed and goal-determined behaviors (Lang & Bradley, 2013), danger really proves to be the stronger cue in some situations, as emphasizing negative consequences has proven to be more powerful than listing positive ones. However, when evaluating decisions regarding sports and vaccination, participants were *more biased* by a positive outcome. In other words participants were partially sensitive to our outcome direction manipulation, depending on the experimental situation. While they consistently judged sports-related, travel-related, and school-related decisions with negative outcomes as worse than decisions without any outcome at all, and decisions without outcomes as worse than decisions with positive outcomes, this difference in evaluations was not observed with vaccination-related decision. Participants evaluated the vaccine-related decision with a negative outcome as having almost the same quality as the decision without outcome: as an extremely low quality decision. The vaccination dilemma was also the only one that did not yield a normatively proposed rational or “correct” answer as a middle position. Taking into account that evaluations of sport-, school-, and travel-related decisions not followed by outcomes were evaluated mostly as neither good nor bad decisions, it seems that those dilemmas do not incorporate a *social* norm defining the “correct” decision, and the answers in those situations followed by outcomes rely more on individual preferences compared to vaccination-related decisions. With vaccination, according to our data, the social norm *should* be and indeed *appears* to be unambiguous, being that not vaccinating a child is a poor quality decision. To summarize, although parents from the sample were not biased when judging the discernment of the fellow parent who made the decision, they failed to produce a normatively correct answer. Accordingly, aside from implying that parents from the sample do not discriminate the decision not to vaccinate with negative outcome from the same decision without outcome, our results indicate that the vaccination dilemma produced a floor effect, which is supported by the ecological rationality notion that sometimes deviations from normatively defined rationality are actually adaptive in environments with particular characteristics (Gigerenzer, 2008, Gigerenzer & Brighton, 2009). It seems that the vaccination context actually served as a facilitator for the adaptive use of biased judgments. A plausible explanation is that because the matter of childhood vaccination is a subject of an ever-growing debate, adaptive behavior might mean choosing a safe position - that is, one’s own (outcome-proof) attitude towards the matter. Even though this finding might seem promising, the positive outcome yielded small, but *observable* OB, indicating that the norm regarding vaccination has been disturbed to some extent rendering its influence weaker in the only possible direction.

Our findings suggest that some decisions, if of particular importance, are not susceptible to the impacts of their respective outcomes. In other words, when parents are judging decisions regarding highly involving health-related decisions, they do not obey the assumptions of proposed descriptive models of reasoning and decision-making. This observation was enabled by the new approach to measuring OB. When faced with descriptions of such important dilemmas with following outcomes and consequences, parents demonstrate less biased judgments of fellow parents’ decisions. Because danger proved to be a strong cue, and because it might be too ambitious to rely on trying to make norms regarding vaccination more solid and unambiguous, when applied to the real world, one of the possible implications of these findings, might be that if we were to combat anti-vaccination movements, those who should be addressing parents are fellow parents, not experts, although further research is needed to fully substantiate this proposition. At the end of the day, the perceived danger cue associated with vaccination, stemming from parent-to-parent communication about “anecdotal cases” is how the anti-vaccination movements themselves have risen in the first place (Arendell, 2000; Smyth & Craig, 2017). Because the ultimate aim of this study was to shed light on the parental decision-making process and its specificities, and perhaps aid the development of customized measures to support parents to overcome biases regarding high-stakes decisions, it’s crucial to point out that insight into what skews their decisions and how, would not be possible with the conventional way of measuring OB. It was only through this addition of the neutral situation to the OB measurement that we were able to give an exact numerical value to the shift in evaluation the parents exhibited, with the “outcome-less” assessment serving as a firm anchor for calculation. Thus, any potential need to make assumptions on the whereabouts of the “true” value of a decision in the parents’ eyes, not influenced by OB, was eliminated.

The main limitation to our study stems from the content of our stimuli. Namely the sports-related item has proven to be more highly involving than we assumed it to be, while the travel-related item had shorter-term consequences (Ariely & Zakay, 2001) compared to the other 3 categories of items. So, in order to claim that being a proxy decision maker makes parents more “irrational” when deciding about children, extending this line of research should incorporate a non-parent control group. Also, it would be desirable to expand the sample used in the potential studies furthering the exploration of this phenomenon, considering the fact that the parents in our sample showed very low bias in highly involving health situations, perhaps somewhat extremely so. This finding would need to be confirmed on a larger sample for us to draw conclusions about the generalizability of the phenomenon to other parents with more certainty. Another avenue of improvement for future research on this topic would be the further refinement and validation of the stimuli used. In other words, providing a higher quantity of stimuli for each of the four combinations of involvement and domain would lend a higher level of credence to any claims made based on the data extracted from the research, both regarding the measurement of outcome bias, and the impact of complexity on its manifestation.

## Appendix

Translation of stimuli, originals were in Serbian.

**Vaccine dilemma** (High Involvement – Health Domain)

*Without outcome:* The doctor has recommended to the parents to get their child vaccinated. The parents are indecisive, because of the debate going on in the media about this subject. They decide not to vaccinate their child.

*Negative outcome*: Later, the child contracted the disease he/she wasn’t vaccinated against, he/she barely managed to recover and it damaged his/her immune system for the rest of his/her life.

*Positive outcome:* The child didn’t contract the disease against which he/she was supposed to be vaccinated and didn’t have any significant health problems throughout his/her life.

**Trip dilemma** (Low involvement – Health Domain)

*Without outcome:* The parents and their child are driving to the seaside. The pediatrician advised them to give the child anti-nausea medicine before the trip, just in case. The parents decide that there is no need for that and set off for the sea.

*Negative outcome:* The child was very sick during the ride and he/she vomited several times.

*Positive outcome:* Everything was fine and the child didn’t get sick during the ride.

**Class dilemma** (High involvement – Non-health Domain)

*Without outcome:* A psychologist advised the parents to enroll their child in the first grade of elementary school, instead of a pre-school kindergarten programme, because, according to his assessment, the child is ready to start school. The parents decide not to enroll their child into the first grade, enrolling him/her in pre-school instead.

*Negative outcome: *Their child is very bored in pre-school and the kindergarten in general, refuses to go to kindergarten and isn’t progressing very well.

*Positive outcome:*Their child is overjoyed about the prospect of spending another year with his/her old friends, can’t wait to go to kindergarten and is still making great progress.

**Sport dilemma** (Low involvement – Non-health Domain)

*Without outcome:* The school councilor advised the parents to sign their child up for a team sport, like volleyball or soccer. They are indecisive, because the child is already taking karate lessons. They decide not to sign up their child for a team sport.

*Negative outcome:* The child kept taking karate lessons, never developing teamwork skills, not learning to cooperate, which presented a problem for him/her in several situations in later life, especially during group activities and tasks.

*Positive outcome:* The child kept taking karate lessons, focused all his/her energy on it, made great progress and developed his/her determination and self-confidence.
